# Intraocular Lens Calculation after Refractive Surgery: A Long-Term Retrospective Comparison of Eight Formulas

**Published:** 2019

**Authors:** David B. Rosen, Madeline B. Heiland, Mitchell Tingey, Harry Y. Liu, Paul Kang, Benjamin Buckner, Yasmyne C. Ronquillo, Phillip C. Hoopes, Majid Moshirfar

**Affiliations:** 1 College of Medicine, University of Arizona, Phoenix, Phoenix, AZ, USA; 2 Hoopes Durrie Rivera Research Center, Hoopes Vision, Draper, UT, USA; 3 McGovern Medical School, Health Science Center, University of Texas, Houston, TX, USA; 4 Mel and Enid Zuckerman College of Public Health, University of Arizona, Phoenix, AZ, USA; 5 John A. Moran Eye Center, Department of Ophthalmology and Visual Sciences, School of Medicine, University of Utah Salt Lake City, UT, USA; 6 Utah Lions Eye Bank, Murray, UT, USA

**Keywords:** Cataract, Refractive Surgery, Intraocular Lens Power, IOL Formula, Linear Mixed Model, IOL Prediction Error

## Abstract

The aim of this study was to compare the accuracy of 8 IOL power calculation formulas for eyes post-refractive surgery. In this Retrospective study, a chart review and data analysis of post-corneal refractive surgery patients who subsequently underwent cataract surgery with IOL implantation in Tertiary surgical center, Draper, UT, USA. The surgery was done in a single surgical center in Draper, UT by one surgeon. The study was approved by the organization’s ethics board. The IOL power formulas used were Barrett True K (BTK), Average Pupil Power (APP), Shammas, Haigis, Galilei, Potvin-Hill Pentacam (PVP), OCT and Barrett True K No History (BTKNH). The percent of time each formula was within ±0.5 D and ±0.75 D of refractive prediction error was calculated. Statistical analysis was performed comparing these 8 methodologies at four post-operative follow-up time points and on the summative time points. Mean follow-up time periods were: 4 weeks, 3 months, 6 months, and 12 months. A total of 64 eyes were included in the study. All IOL formulas showed a myopic trend except APP and Shammas, which showed a hyperopic trend. All tests showed a statistically significant mean absolute value difference from zero. OCT, BTKNH, and BTK had consistently high percentages within ±0.5D and ±0.75 D of refractive error. Linear mixed model analysis showed a statistically significant change in predictive value over time for all formulas. Linear mixed model analysis suggests that it is inadequate to evaluate the performance of IOL power formulae in the short term. Longer-term follow-up is needed to determine accuracy as several factors can result in refractive changes greater than 3 months postoperatively. Our analysis did not demonstrate any formula that was clearly superior to the other methods for predicting IOL power at any time point.

## INTRODUCTION 

Calculating intraocular lens (IOL) power following refractive surgery is a complex process without a single, clear, superior method [1]. Determination of corneal power is integral to IOL power calculation. In the determination of corneal power, a constant called the keratometric index of refraction is used [1]. This constant is adequate if there is a fixed ratio of the anterior and posterior corneal surface curvature. However, in the post-laser-assisted in situ keratomileusis (LASIK) eye, this ratio is disrupted and the above approximation for the keratometric index is flawed [2]. Another difficulty with determining corneal power is that many instruments use the angle of the paracentral cornea to estimate central corneal curvature [1, 3]. Because LASIK reshapes the central cornea, this instrument measurement may be inaccurate [4], and direct measurements of the central cornea curvature are indicated [4]. To overcome these problems, several solutions have been proposed. Some methods rely on both pre and post-LASIK measurements [5-10], while other methods do not require previous measurements [11-18]. The purpose of this study is to compare eight methods for calculating IOL power after corneal refractive surgery.

## METHODS

This is a retrospective chart review of patients with a history of corneal refractive surgery and who subsequently underwent cataract surgery with intraocular lens implantation (IOL). All patients received the ZCB00 lens (Johnson & Johnson Surgical Vision, Inc., Santa Ana, CA, USA) with an A-constant of 119.1 and a Holladay constant of 1.98. The cataract surgeries were performed from August 2016-March 2019 (n=64 eyes) by one surgeon at a single tertiary center, Draper, UT, USA. Patients with a history of corneal refractive surgery, namely: LASIK, photorefractive keratectomy (PRK), and laser subepithelial keratomileusis (LASEK) were included in the study. Patients who had radial keratotomy (RK) were excluded. Time since prior refractive surgery was not included in the data analyzed. There were no exclusions made based on age. Gender and age at the time of cataract surgery and IOL placement were recorded. Data on axial length (AL), anterior chamber depth (ACD), white-to-white (WTW), lens thickness (LT), central corneal thickness (CCT), keratometry (keratometric power in the horizontal [K1] and vertical [K2] planes) were collected for each patient via the LENSTAR LS 900 Software Version i8.2.1.0 (Haag-Streit Diagnostics, Bern, CH). Preoperative manifest refraction and postoperative manifest refraction at 0-2 months (n=56), 2-4 months (n=20), 4-8 months (n=14) and >8 months (n= 20) was recorded. For the remainder of the paper, we will refer to the 0-2 month period as “1 month,” 2-4 month period as “3 months,” 4-8 month period as “6 months,” and >8 month period as “1 year.”

Two methods that required pre-refractive surgery information were used: Barrett True K [19] (BTK) and Average Pupil Power [20] (APP). Six methods that did not require pre-surgical data were used: Shammas [11], Haigis [14], Galilei [1], Potvin-Hill Pentacam [22] (PVP), optical coherence tomography (OCT) based IOL formula [23] and Barrett True K No History [15] (BTKNH). All methods are available at IOLcalc.ascrs.org. The theoretical best (TB) IOL power was calculated using “*TB = Spherical equivalent x 0.7 + implant - target refraction”;* where 0.7 is the ratio between lens power and corneal power such that multiplication of spherical equivalent by 0.7 gives the lens power required to correct refractive error. The absolute and real value difference between each formula and TB was calculated at each time period. Mean age at surgery and means of all biometric data were calculated. Freidman test was used to compare the above 8 IOL formulas at each of the four time intervals. Wilcoxon signed rank test was used to compare significance of mean absolute value difference of each IOL formula (TB-predicted IOL power) from zero. At each time interval, a determination was made of which formula was closest to TB for each patient; if two formulas were equally predictive, both were counted. The number of times an IOL formula was closest to TB was then divided by the number of times that formula was recorded during each specific post-operative period to give a relative yield [ # of times closest to TB/# of times the test was used= relative yield]. The percent of formulas that were within ±0.5 D and ±0.75 D of absolute value of refractive error were calculated. A final summative analysis was done using composite data from all post-operative periods. We termed this the “composite time period” (n=110). Freidman and Wilcoxon signed rank tests were done to compare all groups. P-value of less than 0.05 was used to determine statistical significance. Statistical analysis was done using Stata version 14 software (College Station, TX, USA). Approval was obtained from the Hoopes research committee, and informed consent was signed by each patient. All procedures adhered to the tenets of the Declaration of Helsinki.

## RESULTS

Eyes from 30 men and 34 women were included. The mean± standard deviation (SD) age at the time of IOL implantation was 64.6±8 years old (range 45-79) (Table 1). Among the 64 eyes, the BTK was used 24 times, APP 17 times, Shammas 62 times, Haigis 52 times, Galilei 55 times, PVP 54 times, OCT 53 times, and BTKNH 63 times. In the 1 month time period, mean follow-up was 4 weeks (range 1-8 weeks); for the 3 month time period, mean follow-up was 3 months (range 1-3 months); in the 6 month time period, mean follow-up was 6 months (range 4-8 months);, and at one year, mean follow-up was 12 months (range 8-21 months). All formulas showed a statistically significant mean absolute value difference from zero except APP at the 6-month range (Table 2). In the 1-month range BTK had the lowest mean absolute value difference from TB (0.41±0.33 Diopter [D]), while Galilei had the highest (0.77±0.61 D). At the 1-year range, OCT had the lowest mean absolute value difference from TB (Mean±SD; 0.36±0.27 D), and APP had the greatest (Mean±SD; 0.70±0.49 D). Table 3 shows how often each formula was used at each post-operative period BTKNH was used most often (110 times total), while APP was used least often (27 times total). At the composite time period, both BTK and OCT had a mean absolute difference from TB of 0.48 D. However, BTK had a smaller SD (±0.39) and a narrower range (0.02, 1.415 D) (Table 4). Galilei showed the greatest mean absolute value difference from TB (0.68 ± 0.56 D). On numerical difference from TB, all formulas showed a myopic trend except APP and Shammas, which showed a hyperopic trend. 

**Table 1:** Demographic Data of Study Participants

Variables	Mean Values
Age at Surgery, years (SD)[range];n=64	65 (8) [45,79]
Gender, male (%)	30 (46.9)
Surgery Type, n (%)	
LASIK	57 (89.1)
PRK	5 (7.8)
LASEK	1 (1.6)
LASIK + PRK	1 (1.6)
Average IOL power (SD)[range]; n=64	20.4 (2.2) [16,24.5]
Pre-cataract MRSE (SD)[range]; n=63	-1.4 (2.1) [-8,2.4]
1-month post-cataract MRSE (SD)[range]; n=56	-0.7 (0.7) [-2,1]
3-month post-cataract MRSE (SD)[range]; n=20	-0.4 (0.8) [-2.4-0.75)
6-month post-cataract MRSE (SD)[range]; n=14	-0.5(1.1) [-3.75-1.25]
1-year post-cataract MRSE (SD)[range]; n=20	-0.2(0.7) [-2.25,1]
AL (SD); n=62	25.1 (1.44)
ACD (SD); n=62	3.32 (0.28)
WTW (SD); n=62	12.1 (0.37)
LT (SD); n=61	4.41 (0.39)
K1 (SD); n=62	41.4 (2.31)
K2 (SD); n=62	42.1 (2.44)
CT (SD); n=54	503.9 (41.2)

Abbreviations: SD: standard deviation; n: number; %: percentage; MRSE: manifest refraction spherical equivalent; AL: axial length; ACD: anterior chamber depth; WTW: white to white; LT: lens thickness; K1: horizontal keratometric power in diopters; K2: vertical keratometric power in diopters; CT: corneal thickness; LASIK: laser-assisted in situ keratomileusis; PRK: photorefractive keratectomy; LASEK: laser subepithelial keratomileusis.

**Figure 1:**
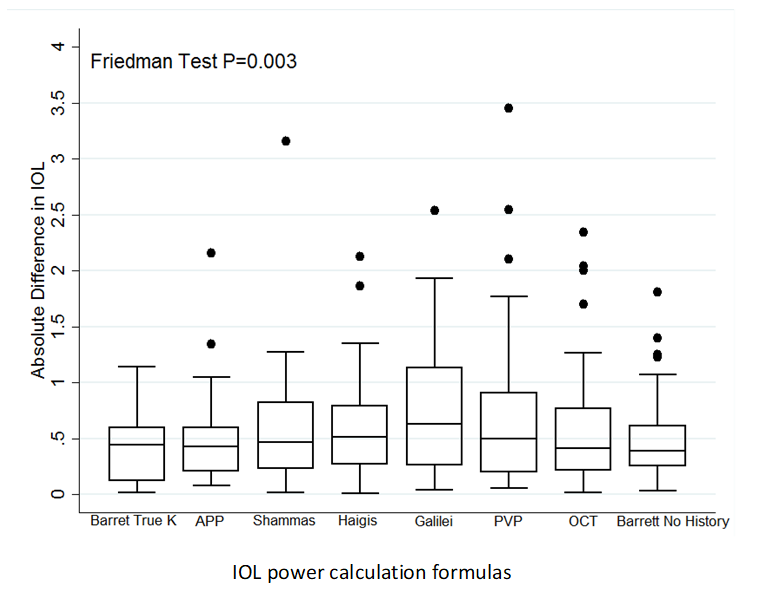
Boxplot of Dioptric IOL Power Prediction Errors with 8 IOL Power Calculation Formulas with Data from the Composite Post-operative Period

Abbreviations: APP: Average Pupil Power; PVP: Potvin-Hill Pentacam; OCT formula: the optical coherence tomography -based IOL power formula; Barrett No History: Barrett True K No History; IOL: Intraocular lens

A Friedman test for data included in the composite time period showed a P value of 0.003 (Fig. 1); however, when analyzing the time points individually only the 1-month range had a significant Friedman Test P value (0.003). Further formula to formula analysis using Wilcoxon signed rank evaluation only showed statistically significant differences were as follows: BTK/Galilei, Shammas/Galilei, Galilei/OCT, and Galilei/BTKNH at composite time period and 1 month; APP/Galilei at composite time period; Haigis/ Galilei at 1 month; Haigis/BTKNH at composite time period; PVP/BTKNH at 1 month (Table 5).

At the composite period, Shammas has the highest relative yield at 26.20%, while BTKNH has the lowest at 7.30% (Table 6). BTK had the highest percentage of predictability within ±0.5 D at 1 month (81.8%), while Galilei had the least (62.0%); however, at the 1-year point, OCT had the highest percentage (94.7%), and APP had the lowest (40.0%) (Table 7). Descending rank order by percent of values within ±0.5 D at the composite time period is as follows: OCT, BTKNH, BTK, Haigis, PVP, APP, Shammas, Galilei. Similar trends were seen within ±0.75 D predictability, with all methods achieving >80% within ±0.75 D on composite time period analysis except the Galilei (78.9%). Descending rank order of tests for predictability within ±0.75 D at the composite time period is as follows: Shammas, BTKNH, OCT, BTK, Haigis, PVP, APP, Galilei. Results of linear mixed model analysis of the mean difference in the absolute value difference of each model over time adjusting for age and gender showed a significant trend with time for all the formulas (Table 8). The reference for this statistical analysis is zero. OCT has the most evident decreasing trend over time.

**Table 2:** Absolute IOL Prediction Error at Various Follow-up Time Periods for Eight IOL Power Calculation Formulas

IOL Prediction Error at Post-cataract Periods	BTK	APP	Shammas	Haigis	Galilei	PVP	OCT	BTKNH
	**Mean (SD)**	**Mean (SD)**	**Mean (SD)**	**Mean (SD)**	**Mean (SD)**	**Mean (SD)**	**Mean (SD)**	**Mean (SD)**
Absolute Difference at 1 month	0.41 (0.33)	0.58 (0.57)	0.55 (0.49)	0.60 (0.46)	0.77 (0.61)	0.69 (0.68)	0.56 (0.53)	0.49 (0.36)
** P-value** ^1^	**<0.001**	**<0.001**	**<0.001**	**<0.001**	**<0.001**	**<0.001**	**<0.001**	**<0.001**
Absolute Difference at 3 months	0.54 (0.35)	0.48 (0.29)	0.55 (0.40)	0.48 (0.38)	0.47 (0.39)	0.76 (0.97)	0.41 (0.31)	0.52 (0.37)
** P-value** ^1^	**0.01**	**0.04**	**<0.001**	**<0.001**	**<0.001**	**<0.001**	**<0.001**	**<0.001**
Absolute Difference at 6 months	0.71 (0.57)	1.07 (0.46)	0.59 (0.41)	0.58 (0.36)	0.65 (0.59)	0.43 (0.28)	0.42 (0.55)	0.53 (0.39)
** P-value** ^1^	**0.04**	0.18	**0.001**	**0.003**	**0.004**	**0.003**	**0.002**	**0.001**
Absolute Difference at 1 year	0.45 (0.46)	0.70 (0.49)	0.48 (0.38)	0.59 (0.28)	0.64 (0.51)	0.69 (0.92)	0.36 (0.27)	0.52 (0.31)
** P-value** ^1^	**0.008**	**0.04**	**<0.001**	**0.002**	**<0.001**	**<0.001**	**<0.001**	**<0.001**

P-values^1^ calculated using the Wilcoxon Signed Rank to determine if the absolute difference is different from zero. P-value less than 0.05 is in bold.

Abbreviations: SD: standard deviation; IOL: Intraocular lens; BTK: Barrett True K; APP: Average Pupil Power; PVP: Potvin-Hill Pentacam; OCT formula: the optical coherence tomography -based IOL power formula; BTKNH: Barrett True K No History.

**Table 3:** Number of Times an IOL Formula was assessed at Each Post-operative Follow-up

Post-cataract	BTK	APP	SHAMMAS	HAIGIS	Galilei	PVP	OCT	BTKNH
1 month (n=56)	22	15	55	45	50	48	46	56
3 months (n=20)	8	5	19	16	17	16	17	20
6 months (n=14)	5	2	14	11	11	11	12	14
1 year (n=20)	9	5	19	13	18	18	19	20
Total (n=64)	44	27	107	85	96	93	94	110

Abbreviations: n: number; IOL: Intraocular lens; BTK: Barrett True K; APP: Average Pupil Power; PVP: Potvin-Hill Pentacam; OCT formula: the optical coherence tomography -based IOL power formula; BTKNH: Barrett True K No History.

**Table 4:** Absolute and Numerical IOL Prediction Error over the Composite Post-operative Period Included for Eight IOL Power Calculation Formulas

	Absolute value prediction error			Numerical value prediction error		
Method	**Mean ± SD**	**Range**	**Median**	**Mean ± SD**	**Range**	**Median**
BTK	0.48 ± 0.39	(0.02,1.42)	0.37	-0.39 ± 0.45	(-1.42,0.24)	-0.37
APP	0.62 ± 0.51	(0.08,2.16)	0.49	+0.32 ± 0.75	(-2.16,1.41)	+0.39
SHAMMAS	0.55 ± 0.45	(0.01,3.16)	0.47	+0.11 ± 0.70	(-3.16,1.32)	+0.16
HAIGIS	0.58 ± 0.41	(0.01,2.12)	0.52	-0.10 ± 0.70	(-2.13,1.49)	-0.03
Galilei	0.68 ± 0.56	(0,2.54)	0.59	-0.13 ± 0.88	(-2.54,2.00)	-0.06
PVP	0.67 ± 0.75	(0.03,3.51)	0.46	-0.51 ± 0.87	(-3.51,0.87)	-0.36
OCT	0.48 ± 0.46	(0.01,2.34)	0.37	-0.13±0.65	(-2.34,1.27)	-0.01
BTKNH	0.51 ± 0.35	(0.02,1.81)	0.43	-0.21±0.59	(-1.81,1.13)	-0.21

Abbreviations: SD: standard deviation; IOL: Intraocular lens; BTK: Barrett True K; APP: Average Pupil Power; PVP: Potvin-Hill Pentacam; OCT formula: the optical coherence tomography -based IOL power formula; BTKNH: Barrett True K No History.

**Table 5:** Wilcoxon Comparative Analysis between Eight IOL Power Calculation Methods at different Time Points

Methods Compared	Composite time period	1 month	3 months	6 months	1 year
	p-values using Wilcoxon signed rank				
BTK=APP	0.26	0.26	0.68	0.18	0.14
BTK=shammas	0.38	0.24	0.86	0.89	0.40
BTK=Haigis	0.18	0.16	0.75	0.22	0.31
BTK=Galilei	**0.02**	**0.01**	0.22	0.18	0.13
BTK=pvp	0.26	0.21	0.50	0.18	0.50
BTK=oct	0.59	0.27	0.31	0.14	0.68
BTK=BTKNH	0.36	0.31	0.16	0.14	0.07
app=shammas	0.43	0.47	0.14	0.65	0.72
app=haigis	0.82	0.82	0.59	0.18	0.59
app=galilei	**0.04**	0.06	0.22	0.65	0.14
app=pvp	0.69	0.86	0.69	0.18	0.50
app=oct	0.43	0.47	0.89	0.18	0.14
app=BTKNH	0.21	0.17	0.89	0.18	0.69
shammas=haigis	0.89	0.99	0.48	0.69	0.25
shammas=galilei	**0.03**	**0.04**	0.62	0.86	0.06
shammas=pvp	0.27	0.21	0.53	0.86	0.24
shammas=oct	0.76	0.75	0.30	0.24	0.63
shammas=BTKNH	0.28	0.29	0.55	0.62	0.66
haigis=galilei	0.06	**0.02**	0.97	0.40	0.92
haigis=pvp	0.62	0.37	0.79	0.94	0.13
haigis=oct	0.92	0.72	0.81	0.21	0.10
haigis=BTKNH	**0.03**	0.08	0.19	0.92	0.16
galilei=pvp	0.18	0.14	0.34	0.29	0.78
galilei=oct	**0.03**	**0.03**	0.80	0.24	0.16
galilei=BTKNH	**0.003**	**0.007**	0.61	0.09	0.19
pvp=oct	0.98	0.44	0.43	0.96	0.46
pvp=BTKNH	0.05	**0.04**	0.38	0.33	0.65
oct=BTKNH	0.46	0.45	0.44	0.39	0.10

Abbreviations: SD: standard deviation; IOL: Intraocular lens; BTK: Barrett True K; APP: Average Pupil Power; PVP: Potvin-Hill Pentacam; OCT formula: the optical coherence tomography -based IOL power formula; BTKNH: Barrett True K No History. P-value less than 0.05 is in bold.

**Table 6:** Relative Yield for each Formula at each Time Period for Eight IOL Power Calculation Formulas

Post-cataract	BTK	APP	SHAMMAS	HAIGIS	Galilei	PVP	OCT	BTKNH
1 month	31.8%	13.3%	25.5%	15.6%	16.0%	18.8%	13.0%	12.5%
3 months	25.0%	0.0%	31.6%	31.3%	17.6%	18.8%	17.6%	5.0%
6 months	20.0%	0.0%	14.3%	9.1%	18.2%	27.3%	41.7%	0.0%
1 year	11.1%	0.0%	36.8%	7.7%	16.7%	27.8%	21.1%	0.0%
Composite time period	25.00%	7.40%	26.20%	15.30%	17.70%	22.60%	19.10%	7.30%

Abbreviations: %: percentage; BTK: Barrett True K; APP: Average Pupil Power; PVP: Potvin-Hill Pentacam; OCT formula: the optical coherence tomography -based IOL power formula; BTKNH: Barrett True K No History.

**Table 7:** Percent of Values within ±0.5D and ±0.75D of Refractive Error

% within ± 0.5 D of refractive error								
Post-cataract	**BTK**	**APP**	**SHAMMAS**	**HAIGIS**	**Galilei**	**PVP**	**OCT**	**BTKNH**
1 month	81.8%	80.0%	72.7%	73.3%	62.0%	62.5%	73.9%	78.6%
3 months	75.0%	80.0%	63.2%	81.3%	82.4%	68.8%	88.2%	70.0%
6 months	60.0%	50.0%	64.3%	81.8%	70.0%	90.9%	91.7%	78.6%
1 year	77.8%	40.0%	68.4%	61.5%	66.7%	83.3%	94.7%	85.0%
Composite time period	77.3%	70.4%	69.2%	74.1%	67.4%	71.0%	83.0%	78.2%
% within ±0.75 D of refractive error								
Post-cataract	**BTK**	**APP**	**SHAMMAS**	**HAIGIS**	**Galilei**	**PVP**	**OCT**	**BTKNH**
1 month	95.5%	80.0%	94.5%	88.9%	72.0%	83.3%	87.0%	94.6%
3 months	100.0%	100.0%	94.7%	87.5%	94.1%	81.3%	94.1%	90.0%
6 months	80.0%	50.0%	85.7%	81.8%	90.0%	100.0%	91.7%	85.7%
1 year	77.8%	80.0%	94.7%	100.0%	77.8%	83.3%	100.0%	95.0%
Composite time period	90.9%	81.5%	93.5%	89.4%	78.9%	84.9%	91.5%	92.7%

Abbreviations: %: percentage; BTK: Barrett True K; APP: Average Pupil Power; PVP: Potvin-Hill Pentacam; OCT formula: the optical coherence tomography -based IOL power formula; BTKNH: Barrett True K No History.

**Table 8:** Results of Linear Mixed Model Analysis of each Method. Linear Mixed Model Ascertains the Mean difference in the Prediction Error (Absolute Difference of IOL) within each Method over Time Adjusting for Age and Gender

	BTK	APP	Shammas	Haigis
	**Beta (95% CI)**	**Beta (95% CI)**	**Beta (95% CI)**	**Beta (95% CI)**
Baseline	**REF**	**REF**	**REF**	**REF**
1 month	0.39 (0.30, 0.49)	0.59 (0.45, 0.72)	0.55 (0.45, 0.66)	0.60 (0.49, 0.71)
3 months	0.51 (0.37, 0.66)	0.51 (0.29, 0.73)	0.52 (0.36, 0.68)	0.49 (0.34, 0.65)
6 months	0.64 (0.46, 0.82)	0.98 (0.64, 1.32)	0.58 (0.40, 0.76)	0.61 (0.43, 0.79)
1 year	0.47 (0.33, 0.61)	0.68 (0.47, 0.91)	0.52 (0.36, 0.68)	0.61 (0.44, 0.78)
Ptrend	**< 0.001**	**< 0.001**	**< 0.001**	**< 0.001**
	**Galilei**	**PVP**	**OCT**	**BTKNH**
	**Beta (95% CI)**	**Beta (95% CI)**	**Beta (95% CI)**	**Beta (95% CI)**
Baseline	**REF**	**REF**	**REF**	**REF**
1 month	0.77 (0.64, 0.89)	0.69 (0.53, 0.84)	0.57 (0.45, 0.68)	0.49 (0.42, 0.58)
3 months	0.53 (0.34, 0.72)	0.70 (0.46, 0.93)	0.43 (0.26, 0.60)	0.48 (0.36, 0.60)
6 months	0.69 (0.15, 0.93)	0.53 (0.25, 0.81)	0.46 (0.26, 0.65)	0.54 (0.39, 0.68)
1 year	0.68 (0.48, 0.87)	0.66 (0.43, 0.88)	0.39 (0.24, 0.56)	0.53 (0.41, 0.65)
Ptrend	**< 0.001**	**< 0.001**	**< 0.001**	**< 0.001**

Abbreviations: CI: Confidence Interval; REF:the reference for this statistical analysis is Zero; %: percentage; IOL: Intraocular lens; BTK: Barrett True K; APP: Average Pupil Power; PVP: Potvin-Hill Pentacam; OCT formula: the optical coherence tomography -based IOL power formula; BTKNH: Barrett True K No History; Ptrend: P value for the trend. P-value less than 0.05 is in Bold.

## DISCUSSION

Numerous studies have sought to assess if there exists a superior formula to determine IOL power for post LASIK and PRK eyes [8, 13, 15, 16, 24]. However, an agreed upon formula does not exist, and some studies have even found that perhaps many formulas perform similarly without significant differences [15, 18]. As the generation of patients who have had corneal refractive surgery increases, many of whom will undergo cataract surgery, this topic has become extremely relevant. The aim of our study was to examine the predictability of the BTK, APP, Shammas, Haigis, Galilei, PVP, OCT and BTKNH in determining lens power in comparison to the calculated theoretical best power. A unique aspect of our study is the 1-year follow-up time (n=20), which provides a reasonable prediction of the formulas’ long-term performance. As demonstrated by the linear mixed model, there is fluctuation of the mean absolute difference from TB over the various follow-up time periods. This fluctuation implies that visual outcomes in the short term are unlikely to persist, and we suggest that evaluation of IOL calculations should include long-term result analysis. Due to the longitudinal retrospective model of our study, we are also able to make a unique statement on the hyperopic and myopic trends of the formulas, which adds dimension to this study. 

We acknowledge that there are limitations of our study and that studies preceding ours have made similar statements regarding the utility of various IOL power calculation formulas. One limitation of our study was the relatively small sample size of data at the 1-year follow-up (n=20), which may reduce the power of our long-term results. Additionally, not all formulas were utilized for each patient, and therefore, some formulas were not applied with adequate frequency. 

In addition, fibrotic changes of the capsule postoperatively may affect Effective Lens Placement (ELP). Subsequently, refractive outcomes may be skewed as ELP can be shifted due to anterior and posterior forces of scarring [25, 26]. This would be yet another factor that could confound our long-term follow-up data as the healing process would influence where the IOL eventually settles in the eye. Our evaluation of APP was limited due to a small sample size (17 eyes with 27 data points over the composite time period). However, a trend towards a hyperopic calculation was noted in this group (Fig. 1). 

Due to our sample size, we do not have enough information to form a recommendation on the use of APP. Galilei was found to have a statistically significant difference when compared to the BTK, Shammas, BTKNH, and OCT formulas at 1 month and over the composite time period. The Galilei had a statistically significant difference compared to APP at the composite time period and compared to Haigis at 1 month (Table 5). Comparing means of Galilei to the means of the other formulas, the data suggest that Galilei may not be as accurate of a predictive method. 

The BTKNH was found to have a statistically significant difference from Haigis at the composite time period and PVP at 1 month. In addition, both the Haigis and PVP had mean absolute differences >0.5 D (Table 4). The Shammas method had no statistically significant differences from any other methods (aside from the previously mentioned comparison to Galilei). However, it trended towards a hyperopic lens calculation. In general, patients tolerate hyperopic shifts in refraction less well than myopic shifts. Therefore, we suggest awareness of this trend when using Shammas to calculate IOL power. The relative yield provides an interesting comparison. While BTKNH had a comparatively low relative yield, the absolute mean difference from TB was one of the closest, with a narrow range, and a high percent within ±0.75 D of refractive error. We interpret this to mean that while the BTKNH rarely predicts closest to the TB, it is consistently very close. In contrast, the PVP had a comparatively high relative yield, but one of the largest absolute mean differences from TB, a wide range, and was only the 6th best for percent within ±0.75 D of refractive error. We interpret this to mean that the PVP either predicts very close to TB or is relatively inaccurate. There were no statistically significant differences between the BTK, BTKNH, and OCT. All had mean absolute differences from TB close to 0.5 D. Both BTK and BTKNH had smaller SD and narrower ranges compared to OCT. OCT had a higher percent yield compared to the BTKNH. It also exhibited the highest percent of outcomes within ±0.5 D of refractive error at all time periods except the 1-month range. However, BTKNH had a higher percent within 0.75 D of refractive error. Given these factors, we cannot say that any one of these three methods is superior. The decision must be made on clinical judgment. Despite this, the BTKNH requires the least amount of equipment and pre-LASIK data. Our results show that BTKNH is not inferior to other methods. Given this, we suggest that the BTKNH is the best option in settings where there is no historical data nor specialized equipment required by some of the other formulas. 

When looking at numerical deviation from TB, Shammas had a slight hyperopic trend while OCT had a slight myopic trend. By averaging the two methods over 91 combined data points, the following were obtained: mean numerical difference from TB 0.01 ± 0.58 D with range of -2.58, +1.175 D. On absolute value difference from TB analysis, mean was 0.43 ± .38 D; range 0.015, 2.58 D; median 0.39 D; percent within ±0.5 D refractive error 86.8% and 95.5% within ±0.75 D (composite time point). While there was no statistically significant difference between this method and the Shammas, BTK, BTKNH, or OCT we suggest that it could be a highly accurate method for predicting IOL power. However, a future prospective study is needed to assess validity. Future study with more data on long term follow up needed to ascertain the magnitude of impact of time on IOL implantation outcomes after refractive surgery. More consistency in follow up with more uniformity on methods used to calculate IOL power could help to establish differences in prediction errors between methods.

## CONCLUSION

As our results show, it can be challenging to select the appropriate IOL power after refractive surgery. New approaches are being taken to more accurately predict the proper IOL; however, the fundamental problems of small sample sizes and lack of long-term follow-up data remain. New fourth-generation IOL formulas show promising results for more accurate prediction[27]. Intra-operative wavefront aberrometry is another method that may be used to calculate IOL power. However, this method appears to produce results of similar accuracy to conventional pre-operative methods [18, 28, 29]. Currently, physicians must carefully weigh input from several formulas and ultimately make a decision based on clinical judgment when selecting IOL power after refractive surgery. We recommend a robust, prospective study examining the utility of fourth- generation formulas and wavefront aberrometry with both six month and one-year follow-up. The data of such a longitudinal study could potentially help fill the current knowledge gap of IOL power formula predictability in a post-refractive surgery patient. 

## DISCLOSURE

Ethical issues have been completely observed by the authors. All named authors meet the International Committee of Medical Journal Editors (ICMJE) criteria for authorship of this manuscript, take responsibility for the integrity of the work as a whole, and have given final approval for the version to be published. No conflict of interest has been presented.

## Funding/Support:

Research to Prevent Blindness, NY, USA
